# Hypertonic Saline for Brain Relaxation and Intracranial Pressure in Patients Undergoing Neurosurgical Procedures: A Meta-Analysis of Randomized Controlled Trials

**DOI:** 10.1371/journal.pone.0117314

**Published:** 2015-01-30

**Authors:** Liujiazi Shao, Fangxiao Hong, Yi Zou, Xiaofang Hao, Haijun Hou, Ming Tian

**Affiliations:** Department of Anesthesiology, Beijing Friendship Hospital, Capital Medical University, Beijing, 100050, P.R. China; Heinrich-Heine University, GERMANY

## Abstract

**Background:**

A wealth of evidence from randomized controlled trials (RCTs) has indicated that hypertonic saline (HS) is at least as effective as, if not better than, mannitol in the treatment of increased intracranial pressure(ICP). However, there is little known about the effects of HS in patients during neurosurgery. Thus, this meta-analysis was performed to compare the intraoperative effects of HS with mannitol in patients undergoing craniotomy.

**Methods:**

According to the research strategy, we searched PUBMED, EMBASE and Cochrane Central Register of Controlled Trials. Other sources such as the internet-based clinical trial registries and conference proceedings were also searched. After literature searching, two investigators independently performed literature screening, quality assessment of the included trials and data extraction. The outcomes included intraoperative brain relaxation, intraoperative ICP, total volume of fluid required, diuresis, hemodynamic parameters, electrolyte level, mortality or dependence and adverse events.

**Results:**

Seven RCTs with 468 participants were included. The quality of the included trials was acceptable. HS could significantly increase the odds of satisfactory intraoperative brain relaxation (OR: 2.25, 95% CI: 1.32–3.81; P = 0.003) and decrease the mean difference (MD) of maximal ICP (MD: −2.51mmHg, 95% CI: −3.39—1.93mmHg; P<0.00001) in comparison with mannitol with no significant heterogeneity among the study results. Compared with HS, mannitol had a more prominent diuretic effect. And patients treated with HS had significantly higher serum sodium than mannitol-treated patients.

**Conclusions:**

Considering that robust outcome measures are absent because brain relaxation and ICP can be influenced by several factors except for the hyperosmotic agents, the results of present meta-analysis should be interpreted with cautions. Well-designed RCTs in the future are needed to further test the present results, identify the impact of HS on the clinically relevant outcomes and explore the potential mechanisms of HS.

## Introduction

Medical management of brain relaxation and intracranial pressure (ICP) is a critical component of anesthesia during the neurosurgical procedures[1,2]. Cerebral swelling(or unsatisfactory brain relaxation) and increased ICP are the commonly clinical situations in patients undergoing craniotomy, which may make the surgical exposure and operative procedure more difficult, and greatly increase the risk of poor outcome if associated with localized cerebral ischemia [2,3]. Thus, there is increasing investigation worldwide exploring the ideal therapy to get satisfactory brain relaxation and ICP during the neurosurgical procedures[4].

It is well-known that various methods including hyperventilation, drainage of cerebrospinal fluid (CSF), and usage of hyperosmotic agents have been used during neurosurgery to control brain relaxation and ICP[4], among which mannitol is considered as the standard and a first-choice hyperosmotic agent for the treatment of increased ICP in a variety of intracranial diseases. However, a wealth of clinical studies have reported that many patients with raised ICP are refractory to the mannitol[5]. And vigorous and repeated administration of mannitol may lead to obvious side effects involving excessive diuresis, electrolyte abnormalities and secondary hypovolemia, while the latter is undesirable in patients undergoing neurosurgical procedures because it may result in intraoperative hypovolemic[6,7]. These issues have led to increasing enthusiasm about the development of alternative approaches in hyperosmotic therapy during neurosurgical procedures.

In recent years, hypertonic saline (HS) has emerged as an attractive alternative in hyperosmotic management to get satisfactory brain relaxation and ICP in a variety of neurosurgical practices[8,9]. A number of prospective clinical trials comparing the effects of HS with mannitol on the ICP have suggested that HS is at least as effective as, if not better than, mannitol in the treatment of intracranial hypertension[10,11]. Moreover, a recent systematic review of HS compared to isotonic solution for peri-operative fluid management indicates HS can reduce the volume of intravenous fluid required to maintain patients undergoing surgery[12]. The intravascular support mechanism of HS may mainly involve its ability to shift fluid from interstitium to intravascular space to counteract perioperative hypotensive effects[8,13]. Thus, in situations such as during neurosurgical procedures where large volume of fluid administration may be accompanied by increased risk of cerebral swelling and intracranial hypertension, a role for HS is emerging. To our knowledge, there is still uncertain about the effects of HS administration in patients undergoing craniotomy compared to mannitol. With the publication of recent literatures involving HS and mannitol[14,15,16,17,18,19,20], it is possible for us to further compare the effects of HS with mannitol during the neurosurgical procedures.

The present meta-analysis was performed to determine the benefits and harms of HS versus mannitol administered to the patients undergoing craniotomy during elective and emergent neurosurgical procedures.

## Materials and Methods

### Study identification

We performed a systematic review of the published literatures to identify all the clinical randomized controlled trials (RCTs), in which HS has been compared to mannitol in the treatment of patients undergoing craniotomy during elective and emergent neurosurgical procedures. Studies that were either not randomized controlled trials or that did not directly involve the effects of HS in patients during neurosurgical procedures were eliminated.

### Search strategy

Based on the text words or MeSH terms such as “Saline Solution, Hypertonic”, “Hypertonic saline”, “Mannitol”, “Neurosurgical Procedures”, and “Craniotomy”, an electronic search for relevant articles was conducted on PUBMED(- present), EMBASE(- present), Cochrane Central Register of Controlled Trials (CENTRAL,—present) and China National Knowledge Infrastructure (CNKI,—present) without language limitation. The internet-based clinical trial registries such as ClinicalTrials.gov, International Clinical Trials Registry Platform (ICTRP) and International Standard Randomized Controlled Trial Number Register (ISRCTN) were also searched for suitable studies. In additional, abstracts and conference proceedings from the Society for Neuroscience in Anesthesiology and Critical Care (SNACC) were searched where available. And we also complemented this by using the *Related Articles* function on PUBMED and searching the reference lists of relevant articles. For full details of the search strategy, see S1 Search Strategy. The search was performed independently by two investigators (Yi Zou and Xiaofang Hao) and was completed on April 2014.

### Literature screening

After literature search, two investigators (Liujiazi Shao and Fangxiao Hong) independently reviewed the titles and abstracts of all studies identified and excluded those that were obviously irrelevant or duplicates. Then the full articles of the remaining studies were retrieved and independently reviewed by them using a structured form to determine eligibility and extract data. Disagreements were resolved by consensus or by a third investigator (Ming Tian) if needed. We contacted study authors for clarifications and further information when necessary.

### Quality assessment

The quality of eligible studies was formally evaluated using the Cochrane Collaboration’s tool for assessing the risk of bias in randomized trials. Specifically, studies were judged on the following items: adequacy of the random sequence generation (“yes” = 2, “unclear” = 1 and “no” = 0); allocation concealment (“yes” = 2, “unclear” = 1 and “no” = 0); blinding (“yes” = 2, “unclear” = 1 and “no” = 0); the incompleteness of outcome data (“yes” = 0 and “no” = 1); the possibility of selective outcome reporting (“yes” = 0 and “no” = 1). The maximum total score is 8. Studies with a total score of <4 were considered as low-quality literatures.

### Data extraction

We extracted the following data from each study: baseline characteristics, the design and objective, number of patients, timing of measurements, main results of the study, and follow-up results. The primary outcomes were intraoperative brain relaxation and intraoperative intracranial pressure. The secondary outcomes included total volume of intravenous fluid, intraoperative diuresis, hemodynamic parameters, electrolyte imbalance, the composite outcome of mortality or dependence for help from other people for their activities of daily living (ADL) at the end of follow-up (at least three months) and adverse events. In the present study, brain relaxation that was assessed on three-, four- or five- point scales would be reported as dichotomized outcome: satisfactory and unsatisfactory. The measurement of intracranial pressure included the one measured through catheterization of the lateral ventricle or CSF pressure through lumbar subarachnoid space.

### Statistical analysis

A meta-analysis based on heterogeneity method was performed using Review Manager for Windows (version 5.2, Cochrane Collaboration and Update Software) for prospective RCTs. Heterogeneity between studies was assessed by means of the standard Cochran Q statistic and I^2^ statistic, which was pre-specified as P<0.10 and I^2^>50% in the present study. The odds ratio (OR) or mean difference (MD) was used as the effect parameter for the meta- analysis and the 95% confidence interval (CI) was used to interpret the results. A fixed-effect model was used to merge the values of OR or MD to estimate the overall effect size when the heterogeneity between studies was not reached. Otherwise, a random-effect model was used in the statistics. All tests were 2-sided and statistical significance was defined as a probability value of <0.05 if not specially stated.

## Results

### Characteristics of included studies

**Fig 1:**
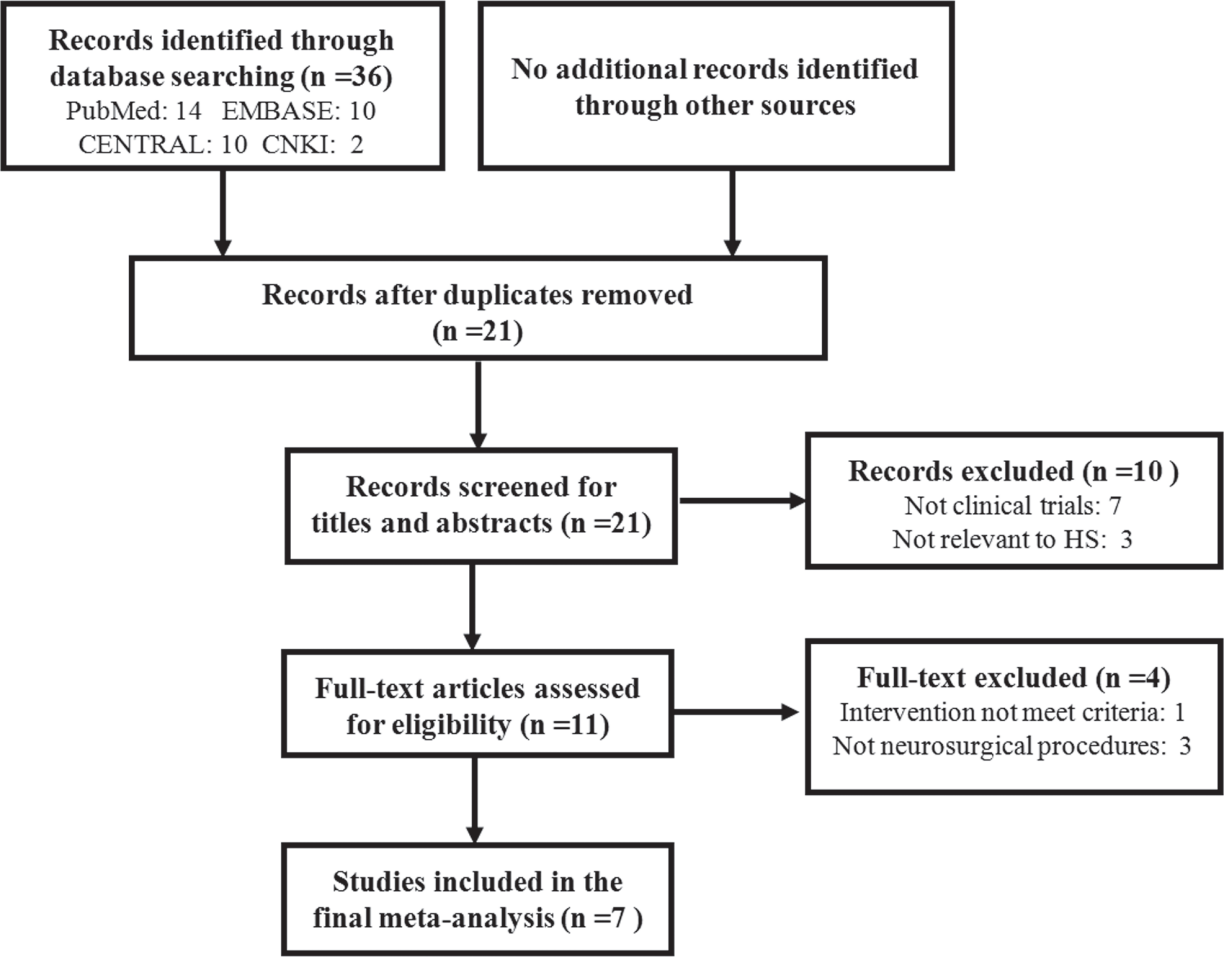
Flow chart of study selection in the present meta-analysis.

In total, 21 articles were initially identified, of which 14 articles were excluded, leaving 7 studies for final analysis. Fig. 1 described the flow chart of searching results and study selection. PRISMA checklist was listed in S1 PRISMA Checklist.

All the seven included studies were prospective RCTs[14,15,16,17,18,19,20]. A total of 468 participants were enrolled in the seven trials, among which 237 (50.6%) patients were included in the group with HS treatment. All trials described the baseline characteristics of the enrolled participants and there were no significant differences in the baseline characteristics of participants between groups in these trials. S1 Table summarized the baseline data of the seven included trials.

As described in S1 Table, the concentration and dose of HS and mannitol studied in the seven included trials varied. The neurosurgical procedures in these trials involved elective neurosurgery[14,15,16,17,20], emergency neurosurgery[18] or both[19]. None of the participants in any of the trials were followed up.

### Risk of bias in included studies

According to the criteria described in the Cochrane Collaboration’s tool for assessing the risk of bias in randomized trials, the risk of bias and quality assessment of these studies was scored and the maximal total score was 8. As described in S2 Table, the score of each item in every study was listed and the total scores of the seven studies ranged from 4 to 7. Overall, the quality of the seven included studies was acceptable.

### Effect of HS on the intraoperative brain relaxation

**Fig 2:**
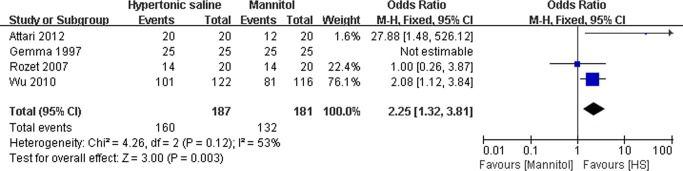
Satisfactory intraoperative brain relaxation in HS and mannitol groups. M-H: Mantel-Haenszel.

As shown in Fig. 2, there were four studies reporting the effect of HS on the intraoperative brain relaxation[14,17,19,20]. In the present study, brain relaxation that was assessed on three-[14,20] or four-[19] point scales in the primary trials was divided as dichotomized outcome: satisfactory and unsatisfactory, which had already been used as measure scale in one study[17]. Data indicated HS could significantly increase the odds of satisfactory intraoperative brain relaxation in comparison with mannitol (OR: 2.25, 95% CI: 1.32∼3.81; P = 0.003), which was associated with statistically non-significant heterogeneity (P = 0.12).

### Effect of HS on the maximal intracranial pressure before opening dura

**Fig 3:**
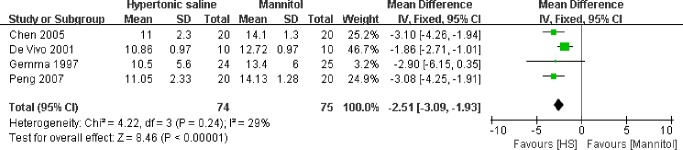
Maximal intraoperative ICP before opening dura in HS and mannitol groups. IV: Inverse Variance.

Data on the effect of HS on the intraoperative ICP was available in four included studies [15,16,17,18](Fig. 3). The ICP in these trials was measured through the catheterization of the lateral ventricle[16] or lumbar subarachnoid space[15,16,17,18]. The pooled mean difference (MD) of maximal ICP before opening dura using HS compared to mannitol was −2.51mmHg (95% CI: −3.39∼−1.93mmHg, P<0.00001), indicating that HS could significantly decrease intraoperative ICP in patients undergoing neurosurgical procedures.

### Effect of HS on the total volume of intravenous fluid and intraoperative diuresis

**Fig 4:**
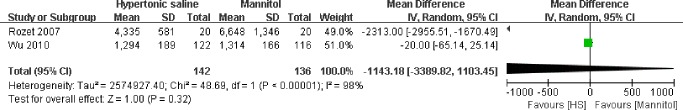
Total volume of intravenous fluid required during neurosurgery in HS and mannitol groups. IV: Inverse Variance.

**Fig 5:**
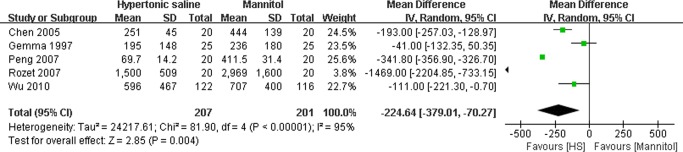
Diuretic output during neurosurgery in HS and mannitol groups. IV: Inverse Variance.

For the total volume of intravenous fluid required and intraoperative diuresis during neurosurgical procedures, data was available in two included studies[19,20] (Fig. 4) and five studies[15,17,18,19,20] (Fig. 5) respectively. Considering that there was statistically significant heterogeneity in the results among the studies (P<0.00001), the random-effect model was used in the statistics. The pooled MD of total volume of intravenous fluid required during operation using HS compared to mannitol was −1143.18ml (95% CI: −338.82∼1103.45ml, P = 0.32)(Fig. 4), which demonstrated that there was no significant difference in total volume of intravenous fluid between the groups treated with either HS or mannitol. However, data on the intraoperative diuresis showed HS could significantly reduce intraoperative urine output during neurosurgical procedures in comparison with mannitol (MD: −224.64ml, 95% CI: −379.01∼−70.27ml; P = 0.004) (Fig. 5).

### Effect of HS on the hemodynamic parameters

**Fig 6:**
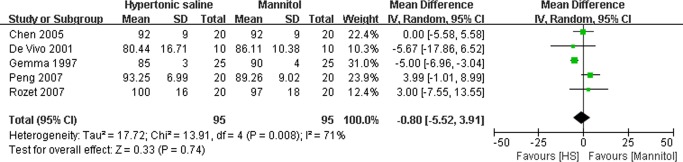
Maximal mean atrial pressure (MAP) in HS and mannitol groups. IV: Inverse Variance.

**Fig 7:**
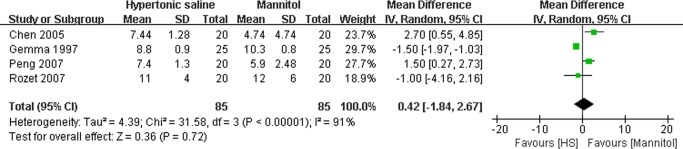
Maximal central venous pressure (CVP) in HS and mannitol groups. IV: Inverse Variance.

As described in Figs. 6 and 7, several trials reported intraoperative hemodynamic parameters such as mean atrial pressure (MAP)[15,16,17,18,19] and central venous pressure (CVP)[15,17,18,19]. Our results indicated there was no significant difference in maximal MAP (MD: −0.80mmHg, 95% CI: −5.52∼3.91mmHg; P = 0.74) (Fig. 6) and maximal CVP (MD: 0.42 mmHg, 95% CI:−1.84∼2.67 mmHg; P = 0.72) (Fig. 7) between the groups treated with either HS or mannitol during neurosurgical procedures.

### Effect of HS on the maximal serum sodium and serum osmolality

**Fig 8:**
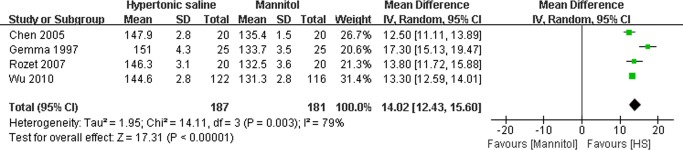
Maximal serum sodium in HS and mannitol groups. IV: Inverse Variance.

**Fig 9:**
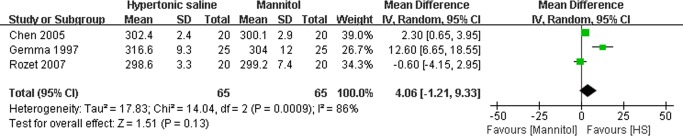
Maximal serum osmolality in HS and mannitol groups. IV: Inverse Variance.

Data on the effects of HS on the maximal serum sodium[15,17,19,20](Fig. 8) and serum osmolality[15,17,19] (Fig. 9) was available in several studies. The pooled MD of maximal serum sodium during neurosurgical procedures using HS compared to mannitol was14.02mmol/L (95% CI: 12.43∼15.60 mmol/L, P<0.00001)(Fig. 8), which demonstrated that HS could significantly increase intraoperative serum sodium in comparison with mannitol in patients undergoing craniotomy. However, data on the maximal serum osmolality indicated there was no significant difference in serum osmolality (MD: 4.06mOsm/L, 95% CI:−1.21∼9.33mOsm/L; P = 0.13) (Fig. 9) between the groups treated with either HS or mannitol during neurosurgical procedures.

### Effect of HS on the mortality or dependence at the end of follow-up (at least three months)

None of the participants in any of the trials were followed up after the end of treatment. Only one trial reported overall mortality at discharge was zero in their study[16]. Assessment of quality of life was not undertaken in any of the trials. Therefore, no trials observed the effect of HS on the composite outcome of mortality or dependence at the end of follow-up.

### Effect of HS on the perioperative adverse events

No serious adverse events such as central pontine myelinolysis, myocardial infarction, cerebrovascular accident and acute kidney failure were reported in these trials.

## Discussion

In the present meta-analysis of seven RCTs, we investigated the intraoperative effects of hypertonic saline compared to mannitol in patients undergoing craniotomy for elective and emergency neurosurgical procedures. The main findings are as follows: (1) HS could significantly increase the odds of satisfactory intraoperative brain relaxation and decrease the mean difference of maximal ICP before opening dura in comparison with mannitol. (2) Compared with HS, mannitol had a more prominent diuretic effect. However, there was no significant difference in total volume of intravenous fluid required between the groups, which was associated with statistically significant heterogeneity. (3) The hemodynamic parameters such as MAP and CVP during surgery were similar in both groups treated with HS or mannitol. (4) Patients treated with HS had significantly higher serum sodium than mannitol-treated patients without differences in serum osmolality between the groups.

It is well-known that to date most of the clinical experiences on the application of HS have come from its use in the traumatic patients, especially in the one with traumatic brain injury[21]. The effects of HS in circulation volume expansion, restoring hemodynamic stability, reducing ICP and possibly improving outcome, provide a basis for the growing interest in its perioperative use over the last two decades[22]. To our knowledge, current studies about the perioperative application of HS are mainly focused on the patients with cardiac surgery[12], in which HS is used to prevent intraoperative hypovolemia and avoid fluid overload during surgery in comparison with isotonic saline. However, there is little known about the difference in the treatment benefit and harms between HS and mannitol for patients with neurosurgical procedures. Thus, our study is aimed to provide a timely and substantial evidence for the clinicians in the selection of appropriate fluid during neurosurgical procedures.

As a critical component of anesthesia during the neurosurgical procedures, good management of brain relaxation and ICP is very important for the neurosurgeons. Satisfactory brain relaxation and ICP can facilitate the surgical access and lesion removal during neurosurgery while cerebral swelling and increased ICP can seriously jeopardize surgical access and increase the risk of poor outcome and neurological deficits. Previous studies involving patients subjected to craniotomy for supra- and infra-tentorial lesions have demonstrated a significant correlation between ICP and the degree of cerebral swelling[3,23]. And it is logical that a tight brain would be expected to occur under conditions of increased ICP when the craniotomy site offers an opportunity for decompression of intracranial structures. Thus well control of intraoperative ICP is beneficial to provide satisfactory brain relaxation during surgery. In this regard, the results of present meta-analysis showed HS could significantly increase the odds of satisfactory intraoperative brain relaxation and decrease intraoperative ICP, which is consistent with the classic theory about the correlation between ICP and brain relaxation.

The conventional explanation of hyperosmolar therapy in reducing ICP is that hyperosmolar solution has the ability to shift fluid from interstitial and intracellular space to intravascular space by establishing the osmotic gradient across intact blood-brain barrier (BBB) in the normal brain tissue. The “dehydration” of the normal brain tissue decreases brain volume, provides additional space for the swelling of injured tissue and will mitigate associated increase in ICP[17]. The effectiveness of the hyperosmolar therapy depends on the “reflection coefficient” (RC) of hyperosmolar solution determining the relative impermeability of the BBB to the solute, where 1 means an absolutely impermeable solute and 0 means an ideally permeable solute. And the RC of HS and mannitol is 1 and 0.9 respectively, which indicates HS is theoretically better than mannitol in establishing the osmotic gradient. However, the effect of hyperosmolar therapy in reducing ICP cannot be predicted simply by its osmolality, since our data showed no difference in serum osmolality between HS and mannitol treatment although there was statistically significant heterogeneity in the results among the included trials. Thus other potential effects of HS, such as improving blood rheology with shrinkage of erythrocytes[24], decreasing production of CSF[11] and anti-inflammatory properties[25,26], may also contribute to its effect in reducing intracranial hypertension together with the hyperosmotic mechanism. Future well-designed RCTs investigating these potential effects of HS versus mannitol are believed to better illustrate these issues.

As we all know, intraoperative fluid management plays an important role in the perioperative anesthesia during neurosurgical practice, which is aimed to maintain fluid balance and hemodynamic homeostasis[4,27]. As the first-choice hyperosmolar solution, mannitol is effective in reducing ICP. However, the side effects of mannitol such as excessive diuresis and secondary hypovolemia, which in turn require larger volume of fluid to maintain hemodynamic homeostasis, are also concerned. As found in the present meta-analysis, mannitol had a more prominent diuretic effect in comparison with HS. Although our data indicated that HS administration was not accompanied with significant reduction in total volume of intravenous fluid required to maintain similar hemodynamic parameters such as MAP and CVP, it was associated with statistically significant heterogeneity. Moreover, only two trials in seven ones reported total volume of intravenous fluid required in their studies and we can find that heterogeneity appears to be due to differences in the magnitude of the effect observed rather than differences in the effect itself. In addition, numerous previous studies have found that HS can significantly reduce the positive fluid balance in patients undergoing surgery in comparison with isotonic saline, which is independent of the type of surgery or perioperative fluid protocol[12]. More trials in the future comparing the effect of HS with mannitol in the intraoperative fluid management may be of benefit to provide more evidence to determine whether HS is accompanied with reduced intraoperative fluid administration in comparison with mannitol.

Considering mortality or dependence is a clinically relevant outcome that is of immediate importance to patients in the context of critical illness such as intracranial diseases, the effects of HS on the mortality or dependence was also studied although most of previous studies involving perioperative HS application are designed to assess its effects in fluid volumes, hemodynamic and biochemical parameters. However, we found no included trials that were designed to measure differences in mortality and none of the participants in any of the trials were followed up after the end of treatment. Thus, neither the included trials nor the present meta-analysis are sufficiently powered to determine the impact of HS on perioperative mortality or morbidity.

Adverse effects associated with hyperosmolar therapy are as important as its efficacy in brain relaxation and ICP during neurosurgery. According to previous numerous reports, electrolyte abnormalities are the most common negative effects[8]. As consistent with previous researches[27], the present meta-analysis also showed that the administration of HS may tend to increase the level of serum sodium early following infusion. A wealth of previous data has suggested the hypernatremia following the infusion of hypertonic saline is transient and serum sodium returns to normal limits by the end of the study especially after single dose of infusion[8,12,27]. Moreover, recent reviews have confirmed that the transient hypernatremia following doses administered in the study is not related to any adverse consequences[11,28,29], which is compatible with that no serious adverse events related to hypernatremia were encountered in the included trials.

There are some limitations in the present meta-analysis. Firstly, although we have attempted to bring together all relevant RCTs in this study and seven RCTs with a total of 468 participants have already been enrolled, a few unpublished researches may be missed and therefore these results may be affected by publication bias. Secondly, generally speaking, pathophysiological endpoints such as brain relaxation and ICP in the present study are more prone to measurement errors or biased reporting compared to the clinical relevant outcomes. In addition, we should keep in mind that either brain relaxation or ICP can also be influenced by several other factors such as patient positioning, craniotomy size, and underlying pathology. However, further subgroup analysis based on these potential factors cannot be performed in present study because individual patient’s data in these included trials are absent. Furthermore, the potential for bias may be further amplified if a number of pathophysiological endpoints are collected in the studies, which may lead to striking treatment effects or even opposite results. Thirdly, there is lack of evidence for explaining the mechanisms of HS in the management of brain relaxation and ICP. The physiological outcome showed in the present study also failed to account for the mechanism. Some potential effects such as anti-inflammatory properties from HS treatment were not described and analyzed in details in this article because these potential effects have not been explored in the RCT studies and only investigated in a few observational studies about the HS. Further trials of high methodology are needed to elucidate these issues in the future.

In conclusion, considering that robust outcome measures are absent in the present meta-analysis because brain relaxation and ICP can be influenced by several factors except for the hyperosmotic agents, the results of present meta-analysis should be interpreted with cautions. Well-designed RCTs in the future are needed to further test the present results, identify the impact of HS on the clinically relevant outcomes and explore the potential mechanisms of HS.

## Supporting Information

S1 PRISMA Checklist PRISMA checklist. (DOC)

S1 Search Strategy Search strategy with details. (DOC)

S1 Table Baseline characteristics of the seven included trials. (DOC)

S2 Table Risk of bias and quality assessment of the seven included trials. (DOC)
