# Supplementary material for: Hypertonic Saline for Brain Relaxation and Intracranial Pressure in Patients Undergoing Neurosurgical Procedures: A Meta-Analysis of Randomized Controlled Trials
Source: PLoS One. 2015 Jan 30;10(1):e0117314. doi: 10.1371/journal.pone.0117314 (PMC4311961; doi:10.1371/journal.pone.0117314)
Supplement: S1 Search Strategy — (DOC) [file pone.0117314.s002.doc]

**Search strategy details**

**PUBMED**

((((((((((clinical trial[Publication Type]) OR (((((((((randomised[Text Word]) OR randomized[Text Word]) OR randomly[Text Word]) OR random order[Text Word]) OR random sequence[Text Word]) OR random allocation[Text Word]) OR randomly allocated[Text Word]) OR at random[Text Word]) OR controlled clinical trial*[Text Word]))) NOT ((((((("Models, Animal"[Mesh]) OR "Animals"[Mesh]) OR "Animal Experimentation"[Mesh]) OR "Animals, Laboratory"[Mesh]) OR "Disease Models, Animal"[Mesh])) NOT humans[MeSH Terms])))))) AND (((("Saline Solution, Hypertonic"[Mesh]) OR (((hypertonic*[Title/Abstract]) OR hyperosmotic*[Title/Abstract]) OR hyperoncotic*[Title/Abstract])))))) AND ((mannitol[MeSH Terms]) OR mannitol[Title/Abstract])) AND (("Neurosurgical Procedures"[Mesh]) OR ((craniotomy[Title/Abstract]) OR neurosurgical[Title/Abstract]) OR neurosurgery[Title/Abstract]))

**EMBASE**

(randomised OR randomized OR randomly OR random AND order OR random AND sequence OR random AND allocation OR randomly AND allocated OR at AND random OR controlled AND clinical AND trial* OR 'clinical trial (topic)'/exp OR 'clinical trial (topic)' NOT ('animal model'/exp OR 'animal model' OR ('animal'/exp OR 'animal' AND ('experiment'/exp OR 'experiment')) OR 'animal'/exp OR 'animal' OR 'experimental animal'/exp OR 'experimental animal' NOT ('human'/exp OR 'human'))) AND ('hypertonic solution'/exp OR 'hypertonic solution' OR hypertonic* OR hyperosmotic* OR hyperoncotic*:ab,ti) AND ('mannitol'/exp OR mannitol) AND ('craniotomy'/exp OR 'craniotomy' OR craniotomy:ab,ti OR neurosurgery:ab,ti OR neurosurgical:ab,ti)

**CENTRAL**

#1 MeSH descriptor: [Saline Solution, Hypertonic] explode all trees

#2 hypertonic*:ti,ab,kw or hyperosmotic*:ti,ab,kw or hyperoncotic*:ti,ab,kw (Word variations have been searched)

#3 #1 or #2

#4 MeSH descriptor: [Mannitol] explode all trees

#5 mannitol:ti,ab,kw (Word variations have been searched)

#6 #4 or #5

#7 MeSH descriptor: [Neurosurgical Procedures] explode all trees

#8 "craniotomy":ti,ab,kw or "neurosurgery":ti,ab,kw or "neurosurgical":ti,ab,kw (Word variations have been searched)

#9 #7 or #8

#10 #3 and #6 and #9
