# Supplementary material for: Hypertonic Saline for Brain Relaxation and Intracranial Pressure in Patients Undergoing Neurosurgical Procedures: A Meta-Analysis of Randomized Controlled Trials
Source: PLoS One. 2015 Jan 30;10(1):e0117314. doi: 10.1371/journal.pone.0117314 (PMC4311961; doi:10.1371/journal.pone.0117314)
Supplement: S1 Table — (DOC) [file pone.0117314.s003.doc]

**Table S1: Baseline characteristics of the seven** included trials

| **Study** | **Groups** | **No.** | **Age(years)** | **Sex(M/F)** | **ASA** | **Neurosurgical procedures** | **Study fluid** | **Dosage** | **Outcomes** |
| --- | --- | --- | --- | --- | --- | --- | --- | --- | --- |
| **Attari 2012** | Intervention | 20 | 48.9±10.8 | n/a | I-III | elective brain tumor surgery | 3.2% HS | 1g/kg | (1) |
|  | Control | 20 | 47.4±12.1 |  |  |  | 20% mannitol |  |  |
| **Chen 2005** | Intervention | 20 | 43±11 | 12/8 | I-II | elective supratentorial glioma surgery | 3% HS | 5.35ml/kg | (2) (4) (5) (6) (7) (8) |
|  | Control | 20 | 41±12 | 11/9 |  |  | 20% mannitol | 1g/kg |  |
| **De Vivo 2001** | Intervention | 10 | 58(17-75) | n/a | I-II | supratentorial cerebral tumor surgery | 3% HS | 3.5ml/kg | (2) (5) |
|  | Control | 10 |  |  |  |  | 18% mannitol | 0.5g/kg |  |
| **Gemma 1997** | Intervention | 25 | 51±14 | 14/11 | I | elective supratentorial lesion surgery | 7.5% HS | 2.5ml/kg | (1) (2) (4) (5) (6) (7) (8) |
|  | Control | 25 | 54±13 | 11/14 |  |  | 20% mannitol | 0.5g/kg |  |
| **Peng 2007** | Intervention | 20 | 37(19-61) | 13/7 | n/a | neurosurgical emergency | 3% HS | 5.33ml/kg | (2) (4) (5) (6) |
|  | Control | 20 | 36(20-65) | 11/9 |  |  | 20% mannitol | 1g/kg |  |
| **Rozet 2007** | Intervention | 20 | 49±13 | 8/12 | II-IV | elective and emergency neurosurgery | 3% HS | 5ml/kg | (1) (3) (4) (5) (6) (7) (8) |
|  | Control | 20 | 48±11 | 7/13 |  |  | 20% mannitol |  |  |
| **Wu 2010** | Intervention | 122 | 56(18-80) | 56/66 | II-III | elective supratentorial brain tumor surgery | 3% HS | 160ml | (1) (3) (4) (7) |
|  | Control | 116 | 54(18-80) | 56/60 |  |  | 20% mannitol | 150ml |  |

**Abbreviation:** n/a: not available; No.: number of patients; ASA: American Society of Anesthesiologists; HS, hypertonic saline; (1): intraoperative brain relaxation; (2):intraoperative intracranial pressure; (3): total volume of intravenous fluid required; (4): intraoperative diuretic output; (5): mean arterial pressure; (6): central venous pressure; (7): serum sodium; (8): serum osmolality.
