# Supplementary material for: Hypertonic Saline for Brain Relaxation and Intracranial Pressure in Patients Undergoing Neurosurgical Procedures: A Meta-Analysis of Randomized Controlled Trials
Source: PLoS One. 2015 Jan 30;10(1):e0117314. doi: 10.1371/journal.pone.0117314 (PMC4311961; doi:10.1371/journal.pone.0117314)
Supplement: S2 Table — (DOC) [file pone.0117314.s004.doc]

| **Study** | **Random sequence generation** | **Allocation concealment** | **Blinding of outcome assessment** | **Incomplete outcome data** | **Selective reporting** | **Total scores** |
| --- | --- | --- | --- | --- | --- | --- |
| **Attari 2012** | unclear(1) | unclear(1) | unclear(1) | no(1) | no(1) | 5 |
| **Chen 2005** | unclear(1) | unclear(1) | unclear(1) | no(1) | no(1) | 5 |
| **De Vivo 2001** | unclear(1) | unclear(1) | unclear(1) | yes(0) | no(1) | 4 |
| **Gemma 1997** | unclear(1) | unclear(1) | yes(2) | no(1) | no(1) | 6 |
| **Peng 2007** | unclear(1) | unclear(1) | yes(2) | yes(0) | no(1) | 5 |
| **Rozet 2007** | unclear(1) | yes(2) | yes(2) | no(1) | no(1) | 7 |
| **Wu 2010** | unclear(1) | yes(2) | yes(2) | no(1) | no(1) | 7 |

**Table S2:** **Risk of bias and quality assessment** **of the seven included trials**

The score of each item is listed in parenthesis.
